# Loop diuretics affect skeletal myoblast differentiation and exercise-induced muscle hypertrophy

**DOI:** 10.1038/srep46369

**Published:** 2017-04-18

**Authors:** Shintaro Mandai, Susumu Furukawa, Manami Kodaka, Yutaka Hata, Takayasu Mori, Naohiro Nomura, Fumiaki Ando, Yutaro Mori, Daiei Takahashi, Yuki Yoshizaki, Yuri Kasagi, Yohei Arai, Emi Sasaki, Sayaka Yoshida, Yasuro Furuichi, Nobuharu L. Fujii, Eisei Sohara, Tatemitsu Rai, Shinichi Uchida

**Affiliations:** 1 Department of Nephrology, Graduate School of Medical and Dental Sciences, Tokyo Medical and Dental University, 1-5-45 Yushima, Bunkyo, Tokyo 113-8519, Japan; 2 Department of Medical Biochemistry, Graduate School of Medical and Dental Sciences, Tokyo Medical and Dental University, 1-5-45 Yushima, Bunkyo, Tokyo 113-8519, Japan; 3 Department of Health Promotion Sciences, Graduate School of Human Health Sciences, Tokyo Metropolitan University, 1-1 Minami-Osawa, Hachioji City, Tokyo 192-0397, Tokyo, Japan

## Abstract

Muscle wasting or sarcopenia contributes to morbidity and mortality in patients with cancer, renal failure, or heart failure, and in elderly individuals. Na^+^-K^+^-2Cl^−^ cotransporter 1 (NKCC1) is highly expressed in mammalian skeletal muscle, where it contributes to the generation of membrane ion currents and potential. However, the physiologic function of NKCC1 in myogenesis is unclear. We investigated this issue using the NKCC1 inhibitors bumetanide and furosemide, which are commonly used loop diuretics. NKCC1 protein levels increased during C2C12 murine skeletal myoblast differentiation, similarly to those of the myogenic markers myogenin and myosin heavy chain (MHC). NKCC1 inhibitors markedly suppressed myoblast fusion into myotubes and the expression of myogenin and MHC. Furthermore, phosphorylated and total NKCC1 levels were elevated in mouse skeletal muscles after 6 weeks’ voluntary wheel running. Immunofluorescence analyses of myofiber cross-sections revealed more large myofibers after exercise, but this was impaired by daily intraperitoneal bumetanide injections (0.2 or 10 mg/kg/day). NKCC1 plays an essential role in myogenesis and exercise-induced skeletal muscle hypertrophy, and sarcopenia in patients with renal or heart failure may be attributable to treatment with loop diuretics.

Muscle wasting or sarcopenia, which is characterized by a progressive decline in skeletal muscle mass and strength^1^, is a common complication that directly contributes to adverse outcomes in various diseases, including cancer, renal failure, and heart failure, and in elderly individuals^1^,^2^,^3^. Its mechanism is complex and poorly understood, but recent studies have revealed that strategies that target and treat sarcopenia can improve patient survival^4^. Therefore, understanding the pathogenesis of the condition may improve its treatment or facilitate the discovery of novel therapeutic options.

Mammalian skeletal muscle exhibits high chloride conductance (GCl)^5^. Previous studies have shown that GCl is significantly reduced in the skeletal muscles of elderly^6^ or uremic^7^ rats. Meanwhile, exercise training increases GCl in rat skeletal muscles^8^. Thus, although the precise mechanisms were not determined in these studies, GCl may be involved in myogenesis, the developmental process through which myoblasts differentiate into myotubes, leading to muscle generation and regeneration^9^. In addition, reduced GCl may be partially responsible for aging- or renal failure-associated sarcopenia. GCl is predominantly attributable to the ClC-1 chloride channel and Na^+^-K^+^-2Cl^−^ cotransporter 1 (NKCC1), which are strongly expressed in mammalian skeletal muscle^10^. Genetic mutations in *ClC-1* causes the dysfunctional skeletal muscle diseases myotonia congenita and myotonic dystrophy^11^, and the differentiation of myoblasts isolated from patients with myotonic dystrophy Type 1 is impaired^12^. However, previous studies on the role of NKCC1 in skeletal muscle have mainly focused on cell volume homeostasis in response to extracellular osmolality^13^, and the physiologic role of NKCC1 in skeletal myogenesis is poorly understood.

In this study, we investigated whether the NKCC1 inhibitors bumetanide and furosemide modulate *in-vitro* differentiation of C2C12 murine skeletal myoblasts and *in-vivo* exercise-induced muscle hypertrophy to clarify the role of NKCC1 in skeletal myogenesis and muscle hypertrophy. This issue has clinical significance because loop diuretics are commonly administered to patients with heart or renal failure and may be associated with sarcopenia in these populations.

## Results

### Na^+^-K^+^-2Cl^−^ cotransporter 1 expression is increased during C2C12 myoblast differentiation

To clarify whether NKCC1 expression changes during the differentiation of C2C12 murine skeletal muscle cells, we examined the expression of total (t) and phosphorylated (p) NKCC1 together with myogenic markers using western blotting.

**Figure 1:**
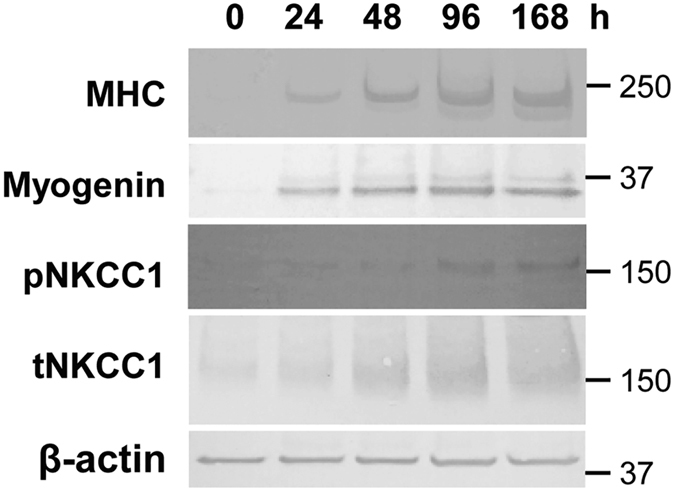
Na^+^-K^+^-2Cl^−^ cotransporter 1 expression is increased during C2C12 murine skeletal myoblast differentiation in parallel with increased expression of the myogenic markers myogenin and myosin heavy chain. MHC, myosin heavy chain; p, phosphorylated; t, total; NKCC1, Na^+^-K^+^-2Cl^−^ cotransporter 1.

In pre-differentiation myoblasts, the expression of muscle-specific proteins myosin heavy chain (MHC) and myogenin—major intermediate and terminal myogenic markers, respectively—were absent or very low (Fig. 1). p/t-NKCC1 expression was similarly low in myoblasts, and was increasingly upregulated during myogenic differentiation in parallel with increasing expression of MHC and myogenin after switching to differentiation medium (DM).

### Inhibition of Na^+^-K^+^-2Cl^−^ cotransporter 1 with bumetanide or furosemide impairs myoblast fusion and the expression of myogenic markers in C2C12 cells

To determine whether NKCC1 inhibition modulates myoblast differentiation and the expression of myogenic marker genes, we measured myoblast fusion index and myotube diameter using immunofluorescence for MHC in differentiating C2C12 skeletal myotubes. Immunoblotting and quantitative reverse-transcription (RT) PCR were performed as well.

**Figure 2:**
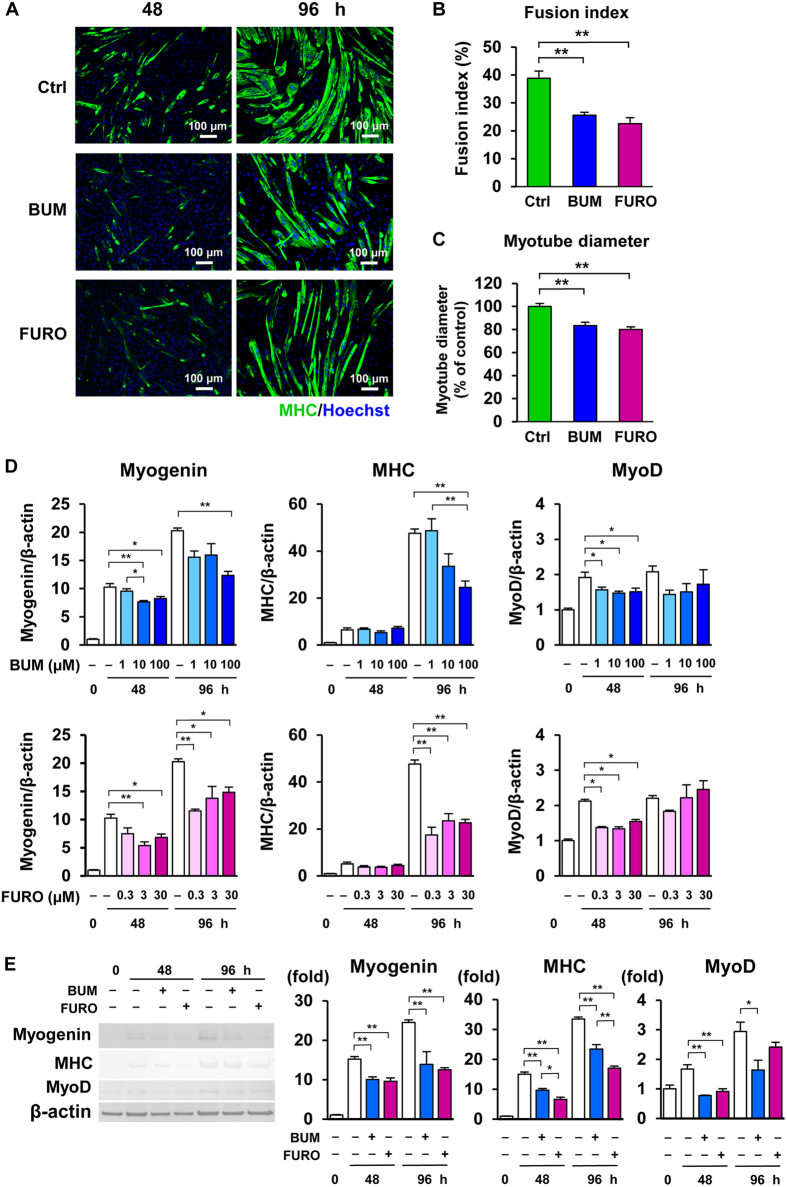
The Na^+^-K^+^-2Cl^−^ cotransporter 1 inhibitors bumetanide and furosemide impair the myogenic differentiation of C2C12 murine myoblasts. (**A**) Immunofluorescence was performed with a myosin heavy chain (MHC) antibody and Hoechst after C2C12 cells were incubated in differentiation medium supplemented with either 10 μM bumetanide (BUM), 3 μM furosemide (FURO), or DMSO alone (Ctrl) for 48 or 96 h. (**B**) The fusion index represents the percentage of multi-nucleated MHC-positive myotubes per vision field after 96 h of differentiation (*n* = 7 independent experiments). Both bumetanide and furosemide markedly suppressed myoblast fusion. (**C**) C2C12 myotube diameters after 96 h of differentiation were significantly lower in bumetanide- and furosemide-treated cells. (**D**) Quantification of MHC, myogenin, and MyoD at 0, 48, and 96 h after treatment with differentiation medium containing bumetanide, furosemide, or DMSO alone by real-time polymerase chain reaction analysis (*n* = 4 per experimental group). mRNA levels were normalized against those of *β-actin*. (**E**) Representative immunoblots of MHC, myogenin, and MyoD, and the densitometric analysis at 0, 48, and 96 h after treatment with differentiation medium containing 10 μM bumetanide, 30 μM furosemide, or DMSO alone (*n* = 3 or 4 per experimental group). Values are presented as the mean ± standard error of the mean. *P < 0.05; **P < 0.01 versus the control group. MHC, myosin heavy chain; BUM, bumetanide; FURO, furosemide; Ctrl, control.

As shown in Fig. 2A, the number of MHC-positive myotubes or multinucleate mature myotubes was markedly decreased with the addition of 10 μM bumetanide or 3 μM furosemide after 96 h differentiation. The calculated fusion index^14^ suggested that the fusion of myoblasts into myotubes, an essential process in myogenesis, was significantly impaired by bumetanide and furosemide compared with control cells (Fig. 2B). The diameters of myotubes were also significantly reduced by bumetanide and furosemide compared with control cells (Fig. 2C).

Quantitative RT-PCR showed that levels of major myogenic marker genes myogenin and MHC were decreased with the addition of 10 or 100 μM bumetanide or 0.3, 3 or 30 μM furosemide 48 h or 96 h after induction with DM (Figs 2D and S1). In accordance with this result, protein expressions of MHC and myogenin were significantly decreased by 10 μM bumetanide or 30 μM furosemide (Fig. 2E). The level of MyoD, a myogenic regulatory factor expressed during the early phase of myogenesis, was significantly decreased by bumetanide and furosemide at 48 h after differentiation, regardless of dose, while the suppression of MyoD was modest at 96 h (Fig. 2D and E). These findings show that bumetanide and furosemide impair myoblast fusion and decrease the expression of myogenic markers, suggesting that NKCC1 has a pivotal role in myogenesis.

We also validated the effect of hydrochlorothiazide (HCTZ), a different class of diuretic drug, on expression of myogenin, MHC, and MyoD by quantitative RT-PCR. As shown in Figure S2, levels of these transcripts were not altered with the addition of 1 or 10 μM HCTZ after 48 and 96 h differentiation. These findings suggest that C2C12 myogenic differentiation was not impaired by HCTZ that does not affect the function of NKCC1.

### Bumetanide and furosemide suppress depolarization-evoked calcium transients in C2C12 myotubes

The possible mechanisms underlying the inhibition of myogenesis by bumetanide and furosemide include a decrease in Ca^2+^ signaling that is one of essential regulators of myoblast differentiation via upregulation of myogenic regulatory transcription factors myogenin and MyoD^15^,^16^. We speculated that the inhibition of NKCC1-mediated GCl affects Ca^2+^ transients following membrane depolarization in skeletal muscle cells similarly as seen in vascular smooth muscle cells^17^,^18^. Thus, we analyzed the effect of bumetanide and furosemide on depolarization-evoked calcium transients after KCl treatment.

Figure S3A shows the mean Fluo-4 (Dojindo Laboratories, Kumamoto, Japan) intensity of five experiments in each group, representing depolarization-evoked calcium transients induced by KCl. The peak Fluo-4 intensity (F-F0) revealed that the Control Group (without bumetanide or furosemide) produced the highest calcium transients (Figure S3B). The NKCC1 inhibitors decreased depolarization-induced Ca^2+^ signaling, which may cause impaired formation of skeletal myotubes.

### Six-week voluntary wheel running upregulates Na^+^-K^+^-2Cl^−^ cotransporter 1 expression in mouse skeletal muscles

The mechanism underlying adult exercise-induced skeletal muscle hypertrophy involves the activation of skeletal muscle satellite cells, their differentiation into myoblasts, and subsequent myoblast fusion into myotubes^19^,^20^. To examine whether NKCC1 expression is altered by chronic exercise training, we analyzed NKCC1 expression in the mouse quadriceps and gastrocnemius muscles after 6 weeks’ voluntary wheel running.

**Figure 3:**
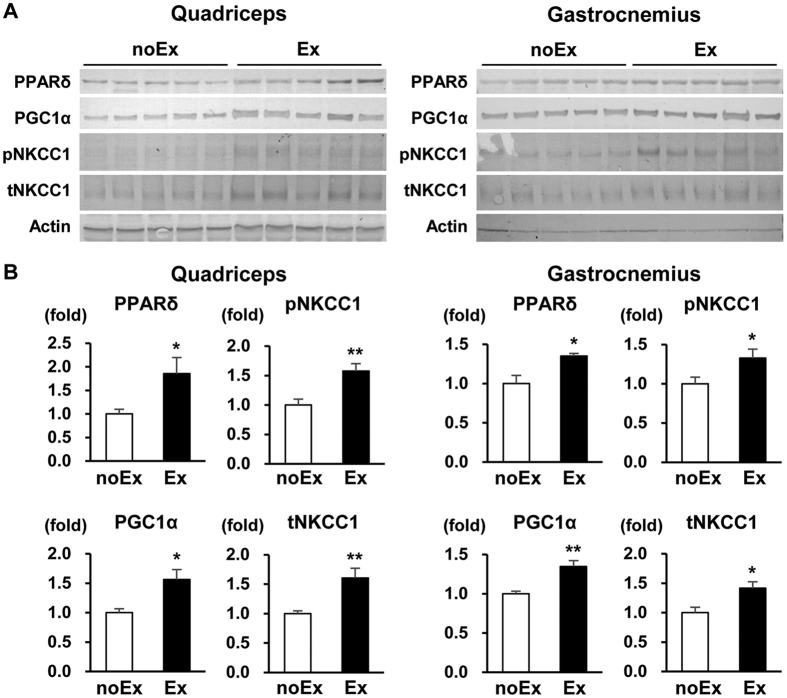
Chronic exercise training upregulates Na^+^-K^+^-2Cl^−^ cotransporter 1 expression in mouse skeletal muscles. (**A**) Immunoblots of PGC1α, PPARδ, total (t), and phosphorylated (p) Na^+^-K^+^-2Cl^−^ cotransporter 1 (NKCC1) in the mouse quadriceps (left) and gastrocnemius (right) muscles after 6-week voluntary wheel running. (**B**) Densitometric analysis of immunoblots of mouse quadriceps (left) and gastrocnemius (right) lysates (*n* = 5 per experimental group). The Exercise (Ex) Group showed higher expression of PGC1α, PPARδ, pNKCC1, and tNKCC1 than the No Exercise (noEx) Group. Values are presented as the mean ± standard error of the mean. *P < 0.05; **P < 0.01 versus the noEx Group. NKCC1, Na^+^-K^+^-2Cl^-^ cotransporter 1; p, phosphorylated; t, total; noEx, No Exercise Group; Ex, Exercise Group.

The running distances within 12 h during the dark–light cycle were measured at 3 and 6 weeks after the initiation of exercise, and showed the acquisition of sufficient running training (Table S1). As shown in Fig. 3, the expression of PPARδ and PGC1α—markers induced by chronic exercise—was significantly increased in both the quadriceps and gastrocnemius muscles after exercise. The Exercise (Ex) Group showed higher expression of p/t-NKCC1 in both muscles compared with the No Exercise (noEx) Group. Thus, we speculated that NKCC1 is also involved in exercise-induced muscle hypertrophy.

### Treatment with daily intraperitoneal bumetanide impairs skeletal muscle hypertrophy induced by chronic exercise training

To further investigate whether NKCC1 is involved in the mechanism underlying *in-vivo* exercise-induced skeletal muscle hypertrophy, we examined the effect of low-dose (0.2 mg/kg/day) and high-dose (10 mg/kg/day) intraperitoneal administration of bumetanide, which provides a lower affinity to NKCC2 with a weaker diuretic efficiency than furosemide, on myofiber sizes after 6 weeks’ voluntary wheel running.

**Figure 4:**
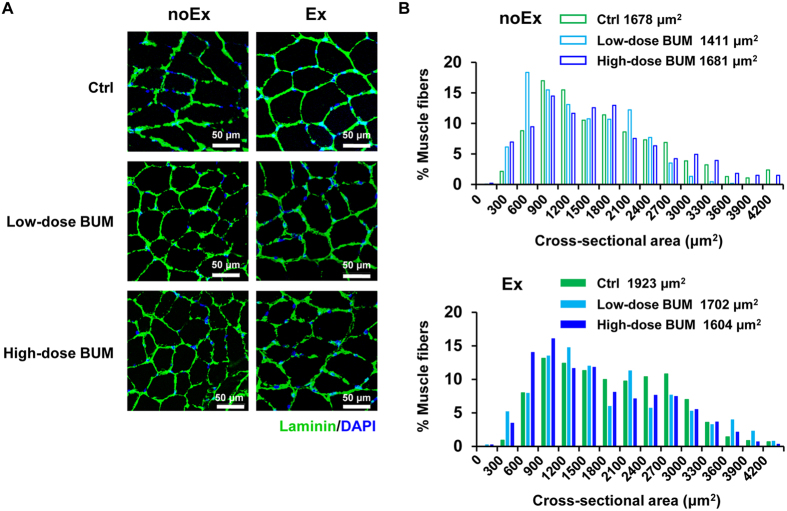
Inhibition of Na^+^-K^+^-2Cl^−^ cotransporter 1 with low- and high-dose bumetanide impairs chronic exercise-induced mouse skeletal muscle hypertrophy. (**A**) Representative immunostaining for laminin in the tibialis anterior muscles after 6-week voluntary wheel running with daily treatment with intraperitoneal low-dose (0.2 mg/kg/day) or high-dose (10 mg/kg/day) bumetanide (BUM) or vehicle (Ctrl). (**B**) Frequency histograms showing the distribution of myofiber cross-sectional area after exercise (Ex, lower) or no exercise (noEx, upper). The noEx Groups showed almost equal distributions independent of bumetanide. The Ex Groups showed increased frequencies of large myofibers compared with the noEx Groups, and the largest increase was seen in the control (Ctrl) group, indicating that both low- and high-dose bumetanide impair chronic exercise training-induced muscle hypertrophy. Values are presented as the median. noEx, No Exercise Group; Ex, Exercise Group; Ctrl, control; BUM, bumetanide.

As shown in Fig. 4A, exercise without bumetanide treatment resulted in a remarkable increase in muscle fiber size after the 6-week experiment. Figure 4B describes the distributions of the cross-sectional area of muscle fibers as well as median values of the histogram (control group with no exercise, 1678 μm^2^; low-dose bumetanide group with no exercise, 1411 μm^2^; high-dose bumetanide group with no exercise, 1681 μm^2^; control group with exercise, 1923 μm^2^; low-dose bumetanide group with exercise, 1702 μm^2^; high-dose bumetanide group with exercise, 1604 μm^2^). Mice without exercise exhibited similar distributions independent of bumetanide treatment. In exercise-trained mice, large myofibers were seen more frequently than mice without exercise, and low- and high-dose bumetanide resulted in fewer large myofibers with a cross-sectional area of approximately 1,800–3,300 μm^2^ compared with mice who received no bumetanide. Low-dose bumetanide did not cause low serum potassium level or increase of blood urea nitrogen, suggesting that this dose did not provide a diuretic efficiency^21^. These results indicate that bumetanide impairs the hypertrophic effect of exercise on skeletal muscle independent of a diuretic effect, and NKCC1 plays an important role in exercise-induced muscle hypertrophy.

### Effect of daily intraperitoneal bumetanide on myofiber central nucleation or fiber type composition after wheel running exercise

To validate if daily intraperitoneal administration of bumetanide also affect skeletal muscle regeneration or myofiber type composition after 6 weeks’ voluntary wheel running, the proportions of myofibers with centrally localized nuclei or those of MHC type I or IIa-positive myofibers were quantified, respectively.

As shown in Figure S4, the numbers of the regenerating myofibers were very limited in the groups without exercise training, and the proportion was not altered by low-dose or high-dose bumetanide treatment. In contrast, the exercise groups showed increase in the numbers of myofibers with central nucleation, and the group untreated with bumetanide showed the largest and significant increase (Figure S4B). Among the three exercise groups, although bumetanide treatment showed a tendency to decrease the numbers of the regenerating myofibers, the differences were not statistically significant. Further examinations with other experimental animal models are needed to clarify the effect of bumetanide on skeletal muscle regeneration.

Myofiber typing revealed that wheel running exercise significantly increased the fraction of MHC type IIa-positive fibers, independent of treatment with bumetanide (Figure S5). In contrast, the analysis of MHC type I showed the equally small proportions of the type I-positive fibers among all the study groups, and neither exercise nor bumetanide influenced this fraction. These findings indicate that bumetanide does not affect the shift in myofiber type composition induced by voluntary wheel running exercise.

## Discussion

In this study, we clarified that NKCC1 expression is upregulated during myogenic differentiation in murine skeletal muscle cells *in vitro* and also after chronic exercise training in mouse skeletal muscle *in vivo*. Moreover, we demonstrated that these alterations in NKCC1 expression play a pivotal role in myogenesis and exercise-induced muscle hypertrophy, based on our findings that the NKCC1 inhibitors bumetanide and furosemide impaired C2C12 myoblast differentiation and mouse muscle hypertrophy after 6 weeks’ voluntary wheel running. These observations provide novel insights into the role of NKCC1 in skeletal muscle physiology, as well as a clinically crucial warning: a large number of patients with renal or heart failure require loop diuretics, and this treatment may contribute to sarcopenia in these populations.

Our *in-vitro* experiments revealed that NKCC1 expression is increasingly upregulated during C2C12 myogenic differentiation in parallel with the increasing expression of myogenic factors. In addition, NKCC1 blockade by bumetanide or furosemide markedly impaired myoblast fusion into multinucleate myotubes, an essential step during skeletal myogenesis associated with muscle generation, regeneration, and hypertrophy in both embryos and adults^9^. To our knowledge, this study is the first to elucidate a physiologic role for NKCC1 in myogenesis. Although bumetanide and furosemide equally impaired myoblast fusion index that is the most reliable myogenic marker (Fig. 2B), furosemide suppressed the myogenic marker genes’ expressions with the lower concentrations than bumetanide and its effect was saturated in the low concentration ranges (Fig. 2D). Furosemide may affect the function of other cation-chloride cotransporters including K^+^-Cl^−^ cotransporters (KCCs) in addition to that of NKCC1^22^. Furthermore, bumetanide and furosemide equally decreased myotube diameters as well (Fig. 2C), and the effect of furosemide was relatively modest compared to its effect on myogenic marker expressions. This may be presumably due to the influences of other various mediators regulating myotube hypertrophy or atrophy *in vitro*.

Furthermore, we showed that bumetanide and furosemide decreased Ca^2+^ transients triggered by KCl-induced depolarization in differentiated myotubes, similarly as seen in vascular smooth muscle cells^17^,^18^, indicating that the inhibition of NKCC1-mediated GCl downregulates depolarization-induced Ca^2+^ signaling. Intracellular Ca^2+^ ([Ca^2+^]_i_) is an essential regulator of myoblast fusion via activation of calcineurin and subsequent upregulation of myogenic regulatory transcription factors MyoD and myogenin^15^,^16^. A recent study involving C2C12 cells showed that silencing of sarcoplasmic/endoplasmic reticulum Ca^2+^ATPases 1b, through which Ca^2+^ is reuptaken into the sarcoplasmic reticulum following Ca^2+^ release via ryanodine receptors, inhibits KCl-induced Ca^2+^ currents, calcineurin activity, and myotube formation^23^. Another study showed that disturbed excitation–contraction coupling and Ca^2+^ homeostasis in human myoblasts impair myoblast differentiation^24^. Therefore, depolarization-induced Ca^2+^ signaling is one of essential regulators of skeletal myogenesis. We speculate that NKCC1 inhibitors affect depolarization-induced Ca^2+^ signaling and subsequent myoblast fusion. However, calcium intensity measured in this method can be affected by various factors such as cell viability, and thus further careful investigations are needed to confirm direct modifications of Ca^2+^ signaling by loop diuretics.

Chronic exercise training by voluntary wheel running upregulated p/t-NKCC1 expression together with the expression of the established exercise-induced markers PPARδ and PGC1α in mouse skeletal muscle *in vivo*. A previous study on exercise-mediated potassium regulation involving NKCC1 in rat skeletal muscles showed that increased ^86^Rb uptake occurs via NKCC1, indicating that NKCC1 activity is increased in combination with increased t-NKCC1 expression after 4-week treadmill training^8^. The alteration in p/t-NKCC1 expression after long-term exercise in our study was compatible with these findings, and let us to investigate whether exercise-induced hypertrophy can be modulated by daily intraperitoneal bumetanide administration during the exercise period. Our results showed that chronic exercise increased the population of large myofibers, and that this hypertrophic effect of exercise was suppressed by low- and high-dose bumetanide independent of its diuretic effect. Chronic exercise is known to activate skeletal muscle satellite cells that differentiate into myoblasts and fuse into established myotubes, eventually resulting in muscle hypertrophy^19^,^20^. Therefore, the impediment of *in-vitro* C2C12 myoblast fusion by bumetanide theoretically corresponds to that of *in-vivo* exercise-induced muscle hypertrophy by bumetanide.

In conclusion, NKCC1 expression is increased in differentiating C2C12 cells and in mouse skeletal muscle after exercise training, and NKCC1 inhibition with bumetanide or furosemide impairs myogenic differentiation and exercise-induced muscle hypertrophy. Loop diuretics that are commonly administered to patients with heart failure or kidney failure may be involved in the pathogenesis of sarcopenia in these populations.

## Methods

### Cell culture

Murine C2C12 skeletal myoblast cells were cultured in DMEM (Invitrogen, Carlsbad, CA, USA) supplemented with 10% fetal bovine serum, 4 mM l-glutamine, 100 U/mL penicillin, and 0.1 mg/mL streptomycin at 37 °C and 5% CO_2_ in a humidified incubator. After C2C12 myoblasts reached 70–80% confluence, differentiation was initiated by replacement of the culture medium with DM: DMEM containing 2% horse serum (Sigma-Aldrich Corp., St. Louis, MO, USA). DM was supplemented with either bumetanide (Sigma-Aldrich Corp.; 1, 10, or 100 μM) or furosemide (Sigma-Aldrich Corp.; 0.3, 3, or 30 μM) dissolved in DMSO (equally consisting of 0.1% DMSO in DM), or DMSO alone. The respective concentrations of bumetanide and furosemide were selected based on the previous studies using these drugs in cultured cells as well as their different affinities to NKCC1^25^,^26^. The cells were additionally incubated for 2–7 days until each experiment.

### Animals and treatment

All experiments were performed in accordance with the guidelines for animal research of Tokyo Medical and Dental University, and the protocol was approved by The Animal Care and Use Committee of Tokyo Medical and Dental University. Male C57BL/6 J mice (7 or 8 weeks old at arrival) were fed normal chow and water under standard lighting conditions (12-h:12-h light–dark cycle). On arrival, mice were randomly assigned to either the Ex or noEx Groups. Voluntary wheel running was performed for 6 weeks in a plastic cage (height, 140 mm; width, 215 mm; depth, 320 mm), and the Ex Group mice were given free access to a running wheel (wheel circumference, 140 mm; Melquest, Toyama, Japan). Daily revolutions of this wheel were counted with a magnetic counter attached to the wheel.

The experiments to validate the effect of bumetanide on exercise-induced muscle hypertrophy were performed by a combination of 6-week voluntary wheel running with daily intravenous injections of either low-dose bumetanide (Sigma-Aldrich Corp.; 0.2 mg/kg body weight), high-dose bumetanide (10 mg/kg body weight), or vehicle (0.9% saline) equally containing 40% DMSO (10 mL/kg body weight). The low dose of 0.2 mg/kg was selected given that this dose was sufficient dose even for brain penetration in various neurologic rodent models^27^,^28^, and did not induce a diuretic efficiency that can cause dehydration and modulate muscle functions^21^.

After 6 weeks’ exercise with and without bumetanide or vehicle, the mice were euthanized, and the quadriceps, gastrocnemius, and tibialis anterior muscles were removed for protein extraction and immunofluorescence.

### Immunoblotting

C2C12 myoblasts or differentiated myotubes grown in a 6-well plate were lysed in RIPA buffer (50 mM Tris-HCl [pH 7.5], 150 mM NaCl, 0.5% sodium deoxycholate, 0.1% SDS, 1 mM EGTA, 1 mM EDTA, 1 mM sodium orthovanadate, 50 mM sodium fluoride, 1% Triton X-100, and Protease Inhibitor Cocktail [Roche Diagnostics, Basel, Switzerland]) for 30 min at 4 °C. The lysates were centrifuged at 12,000 × *g* for 5 min, and the supernatants were diluted with 2 × SDS sample buffer (Cosmo Bio USA Co., Carlsbad, CA, USA) and denatured at 60 °C for 20 min. Muscle homogenates were lysed in lysis buffer as previously described^29^ and the lysates were centrifuged at 17,000 × *g* for 10 min. After centrifugation, the supernatants were diluted with 2 × SDS and denatured similarly.

The following primary antibodies were used: rabbit anti-phosphorylated NKCC1 (T206; 1:300)^30^; mouse anti-NKCC1 (T4; Developmental Studies Hybridoma Bank, University of Iowa, Iowa City, IA, USA; 1:5,000); mouse anti-myogenin (F5D; Santa Cruz Biotechnology, Inc., Dallas, TX, USA; sc-12732; 1:500); rabbit anti-MyoD (Santa Cruz Biotechnology, Inc., Dallas, TX, USA; sc-760; 1:200); mouse anti-MHC (A4.1025; Upstate Biotechnology Inc., Lake Placid, NY, USA; 1:1,000); and rabbit anti-Actin (Cytoskeleton, Inc., Denver, CO, USA; 1:250). Alkaline phosphatase-conjugated anti-Immunoglobulin G antibodies (Promega Corp., Madison, WI, USA) were used as secondary antibodies. The band densities of the proteins were quantified using ImageJ (National Institutes of Health, Bethesda, MD, USA). Immunoblots of total NKCC1 in C2C12 myotube and various tissues^31^ including skeletal muscle are described in Figure S6, suggesting that the used antibody specifically detects this protein both in myotubes and skeletal muscles.

### Quantitative real-time reverse-transcription polymerase chain reaction

Total RNA was extracted from C2C12 myoblasts or myotubes using the SingleShot™ Cell Lysis Kit (Bio-Rad Laboratories, Inc., Hercules, CA, USA) according to the manufacturer’s protocol. cDNA was synthesized using ReverTra Ace^®^ (Toyobo Co., Ltd., Osaka, Japan). Quantitative real-time PCR was performed on a Thermal Cycler Dice Real Time System Lite TP700 (Takara Bio Inc., Otsu, Shiga, Japan) using SYBR^®^ Premix Ex Taq™ II (Takara Bio Inc.). The primers used in this study are shown in Table S2. PCR amplification consisted of 40 cycles (95 °C for 5 s, 60 °C for 30 s) after an initial denaturation step (95 °C for 30 s). All reactions were performed in duplicate, and the relative mRNA expression level of each target gene was normalized with that of *β-actin* as an internal control. In addition, the 2^−ΔΔct^ method^32^ was used to compare changes in mRNA expression levels.

### Immunofluorescence

C2C12 cells 48 and 96 h after the induction of differentiation were fixed in 3% paraformaldehyde in PBS for 15 min, blocked with 1% BSA in PBS for 30 min, and incubated in 0.2% Triton X-100 in PBS for 10 min. After three washes in PBS, the cells were treated with mouse anti-MHC antibody (A4.1025; Upstate Biotechnology Inc.; 1:200) in PBS supplemented with 0.1% BSA for 4 h at room temperature. After three washes in PBS, the cells were incubated with 488 goat anti-mouse antibody (Molecular Probes, Eugene, OR, USA; A-11029; 1:200) with Hoechst 33342 for 1 h, washed three times in PBS, and analyzed using an IX71 CCD microscope (Olympus Corp., Tokyo, Japan).

We calculated the fusion index^14^, representing the percentage of MHC-positive myotubes with ≥ two nuclei of the total myotubes within each field, to quantify myoblast fusion. To analyze myotube diameter, we took pictures from each well and all obtained myotubes (>200 per each experimental group) were included in the analysis. Each diameter of a single myotube was determined using ImageJ.

Immediately after removal, fresh tibialis anterior muscle was covered with OCT compound and snap-frozen in semi-frozen isopentane with liquid nitrogen for approximately 30 s. Frozen sections 15-μm thick were mounted on glass slides and fixed in 4% paraformaldehyde. Immunofluorescence staining of these sections was performed with both anti-laminin polyclonal antibodies (Sigma-Aldrich Corp.; L-9393; 1:200) and anti-DAPI antibodies. The cross-sectional area of the myofibers was analyzed by confocal microscopy using ImageJ. Myofiber numbers and percentages of myofibers with centralized nuclei were also analyzed to quantify the proportion of regenerating myofibers^14^. Moreover, muscle fiber typing was performed with antibodies against MHC type I (M8421, Sigma-Aldrich Corp)^33^ and MHC type IIa (SC-71, Developmental Studies Hybridoma Bank).

### Calcium imaging

C2C12 cells were differentiated for 96 h in DM in glass-bottomed dishes. Differentiated myotubes were loaded with the Ca^2+^ indicator Fluo-4 (5 μg/mL) supplemented with 10 μM bumetanide, 3 μM furosemide, or DMSO alone at 37 °C and 5% CO_2_ for 1 h. Depolarization was induced with 120 mM KCl, and the relative fluorescence intensity, indicating changes in intracellular Ca^2+^ concentration, were monitored using confocal microscopy.

### Statistics

Statistical significance was evaluated using the unpaired *t* test for two groups, or by one-way analysis of variance (ANOVA), followed by Bonferroni’s test for multiple groups. All data are presented as the mean ± standard error of the mean, and *P* < 0.05 was considered statistically significant.

## Additional Information

**How to cite this article:** Mandai, S. *et al*. Loop diuretics affect skeletal myoblast differentiation and exercise-induced muscle hypertrophy. *Sci. Rep.* **7**, 46369; doi: 10.1038/srep46369 (2017).

**Publisher's note:** Springer Nature remains neutral with regard to jurisdictional claims in published maps and institutional affiliations.

## Supplementary Material

Supplementary Information
