# Supplementary material for: Loop diuretics affect skeletal myoblast differentiation and exercise-induced muscle hypertrophy
Source: Sci Rep. 2017 Apr 18;7:46369. doi: 10.1038/srep46369 (PMC5394462; doi:10.1038/srep46369)
Supplement: Supplementary Information [file srep46369-s1.pdf]

Online Supplement

Loop diuretics affect skeletal myoblast differentiation and exercise-induced muscle hypertrophy

Shintaro Mandai[1], Susumu Furukawa[1], Manami Kodaka[2], Yutaka Hata[2], Takayasu Mori[1], Naohiro Nomura[1], Fumiaki Ando[1], Yutaro Mori[1], Daiei Takahashi[1], Yuki Yoshizaki[1], Yuri Kasagi[1], Yohei Arai[1], Emi Sasaki[1], Sayaka Yoshida[1], Yasuro Furuichi[3], Nobuharu L. Fujii[3], Eisei Sohara[1], Tatemitsu Rai[1], and Shinichi Uchida[1]*

[1]Department of Nephrology, Graduate School of Medical and Dental Sciences, Tokyo Medical and Dental University, 1-5-45 Yushima, Bunkyo, Tokyo 113-8519, Japan

[2]Department of Medical Biochemistry, Graduate School of Medical and Dental Sciences, Tokyo Medical and Dental University, 1-5-45 Yushima, Bunkyo, Tokyo 113-8519, Japan

[3]Department of Health Promotion Sciences, Graduate School of Human Health Sciences, Tokyo Metropolitan University, 1-1 Minami-Osawa, Hachioji City, Tokyo 192-0397, Tokyo, Japan

*Correspondence to: Shinichi Uchida

Department of Nephrology, Graduate School of Medical and Dental Sciences, Tokyo Medical and Dental University
1-5-45 Yushima, Bunkyo, Tokyo 113-8519, Japan
Tel: +81-3-5803-5214; Fax: +81-3-5803-5215; E-mail: suchida.kid@tmd.ac.jp

Supplementary Figure 1. Relative expression levels of myogenin, myosin heavy chain, and MyoD mRNA by real-time polymerase chain reaction analysis and the $2^{-\Delta\Delta ct}$ method.

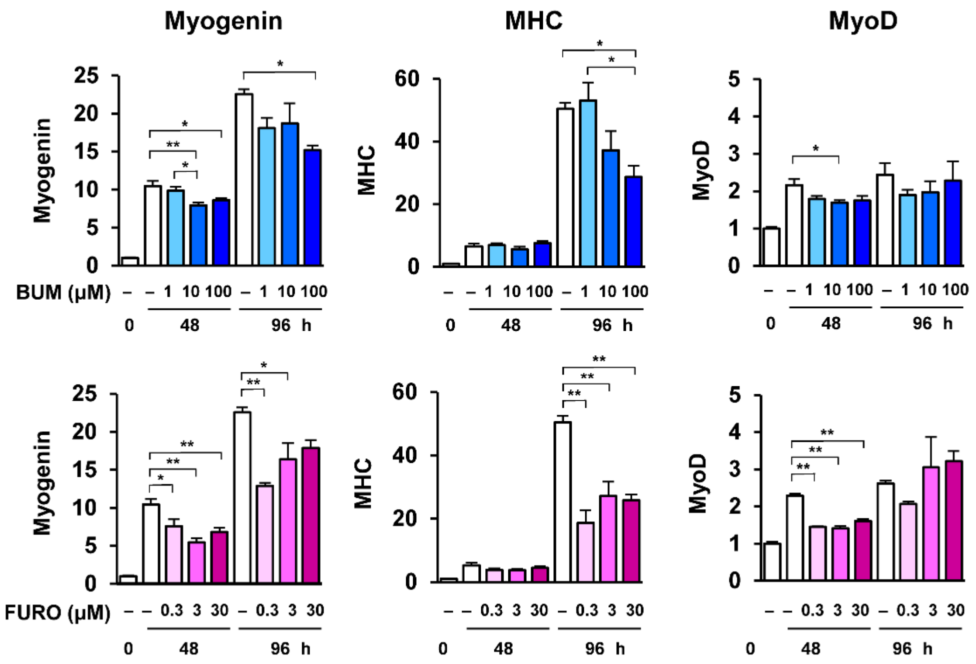

Quantification of MHC, myogenin, and MyoD at 0, 48, and 96 h after treatment with differentiation medium containing bumetanide, furosemide, or DMSO alone by real-time polymerase chain reaction analysis (n = 4 per experimental group). Values are presented as the mean ± standard error of the mean. *P < 0.05; **P < 0.01 versus the control group. MHC, myosin heavy chain; BUM, bumetanide; FURO, furosemide; Ctrl, control.

Supplementary Figure 2. Hydrochlorothiazide does not affect C2C12 myoblast differentiation.

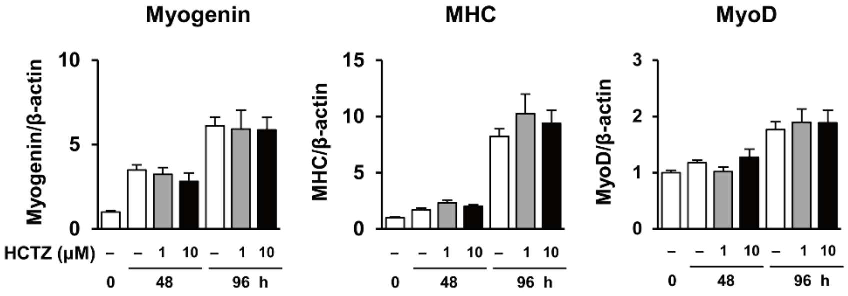

Quantification of myogenin, myosin heavy chain (MHC), and MyoD transcripts by real-time polymerase chain reaction analysis at 0, 48, and 96 h after C2C12 cells were incubated in differentiation medium supplemented with either 1 or 10 μM hydrochlorothiazide (HCTZ), or DMSO alone (n = 4 per experimental group). mRNA levels of these transcripts normalized against those of *β-actin* were not significantly altered with HCTZ. Values are presented as the mean ± standard error of the mean. MHC, myosin heavy chain; HCTZ, hydrochlorothiazide.

Supplementary Figure 3. Depolarization-evoked calcium transients are reduced in differentiated C2C12 myotubes by bumetanide and furosemide.

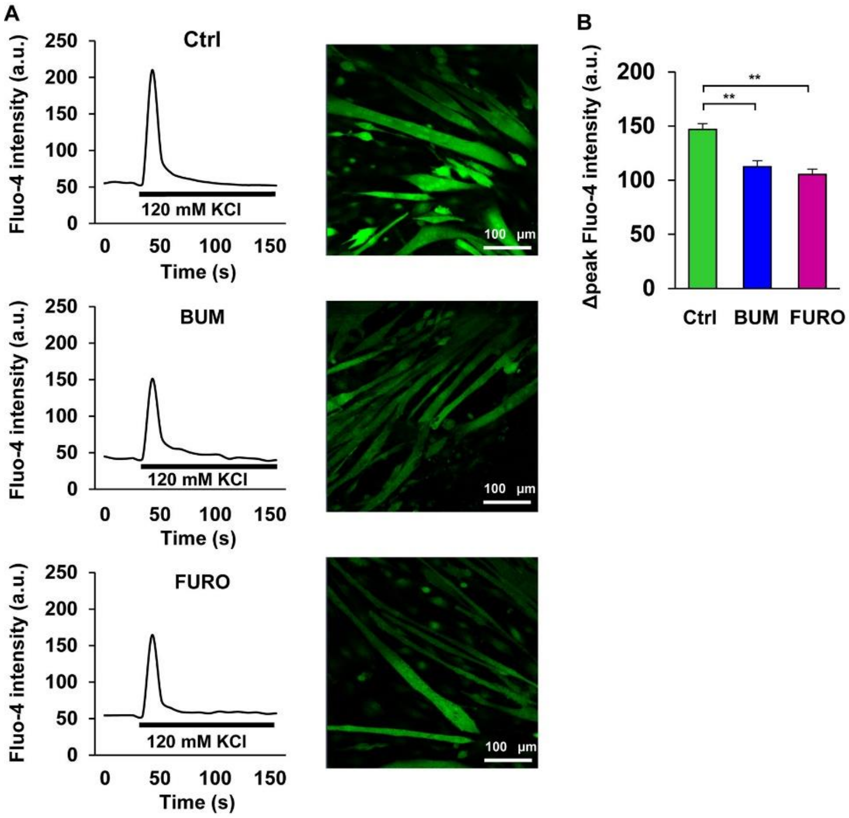

A, Averaged records of Fluo-4 intensity in response to 120 mM KCl in C2C12 myotubes treated with 10 μM bumetanide (BUM), 3 μM furosemide (FURO), or DMSO alone (Ctrl) from five experiments (left), and representative images showing Fluo-4 peak intensity, respectively (right). **B,** Δpeak Fluo-4 intensity (F-F0) among the groups (*n* = 10 or 15 per experimental group). Values are presented as the mean ± standard error of the mean. $^{**}P < 0.01$ versus the control group. Ctrl, control; BUM, bumetanide; FURO, furosemide.

Supplementary Figure 4. Effect of bumetanide administration on proportions of mouse muscle fibers with central nucleation after voluntary wheel running.

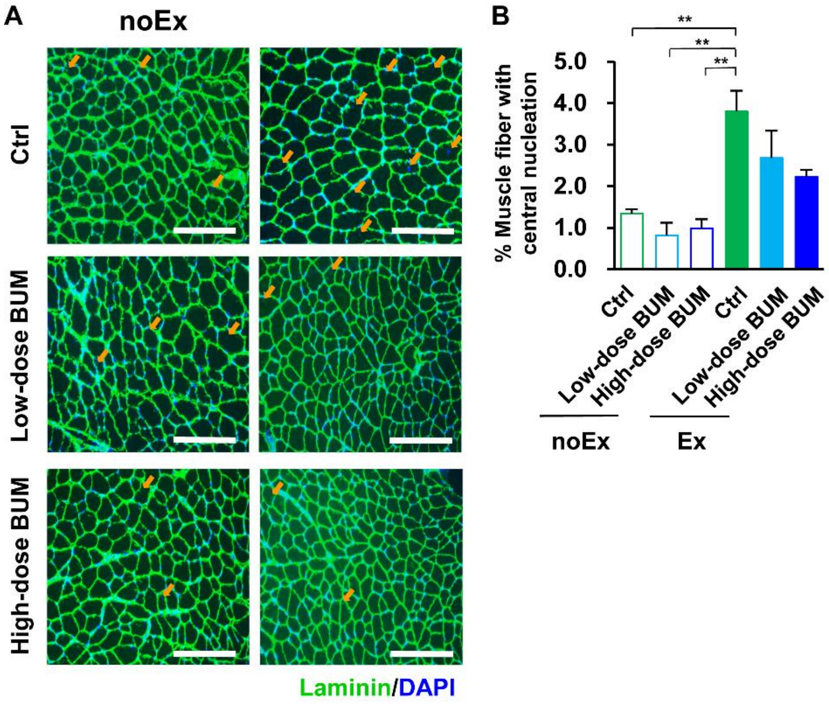

A, Representative immunostaining for laminin and nuclei in the tibialis anterior muscles of mice after 6-week voluntary wheel running with daily treatment with intraperitoneal low-dose (0.2 mg/kg/day) or high-dose (10 mg/kg/day) bumetanide (BUM) or vehicle (Ctrl). Arrows indicate non-peripherally or centrally localized nuclei in myofibers. Scale bars, 200 μm. **B,** Proportion of myofibers with central nucleation in each experimental group. Values are presented as the mean ± standard error of the mean (n = 3 or 4 for each group). $^{**}P < 0.01$. Ctrl, control; BUM, bumetanide.

Supplementary Figure 5. Changes in myofiber composition in mice with bumetanide administration after voluntary wheel running exercise.

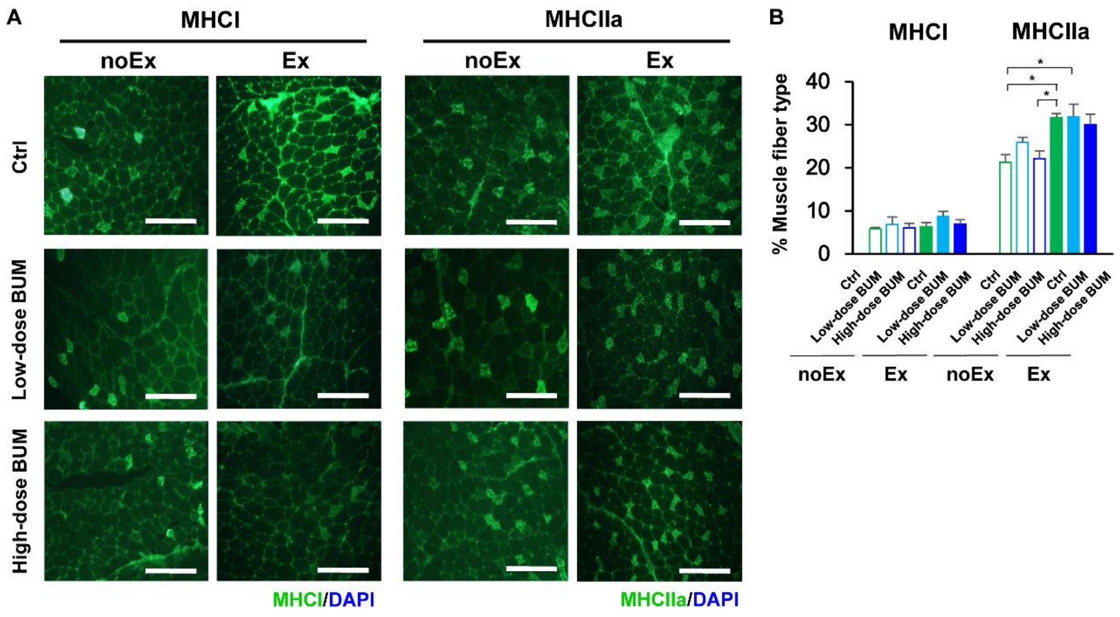

A, Indirect immunofluorescence staining of MHC type I or type IIa in the tibialis anterior muscle sections from mice after 6-week voluntary wheel running with daily treatment with intraperitoneal low-dose (0.2 mg/kg/day) or high-dose (10 mg/kg/day) bumetanide (BUM) or vehicle (Ctrl). Scale bars, 200 µm. **B,** Quantitative fiber type analysis of the muscles. Values are presented as the mean ± standard error of the mean (n = 3 or 4 for each group). *$P < 0.05$. Ctrl, control; BUM, bumetanide.

Supplementary Figure 6. Immunoblots of total Na–K–Cl cotransporter 1 in murine C2C12 myotube and various mouse tissues.

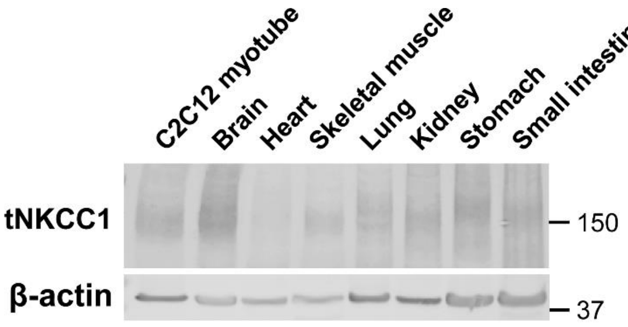

Immunoblots of total (t) Na–K–Cl cotransporter 1 (NKCC1) in murine C2C12 myotube and various tissues from a wild-type mouse (30 μg per lane for each tissue lysate).

Supplementary Figure 7.

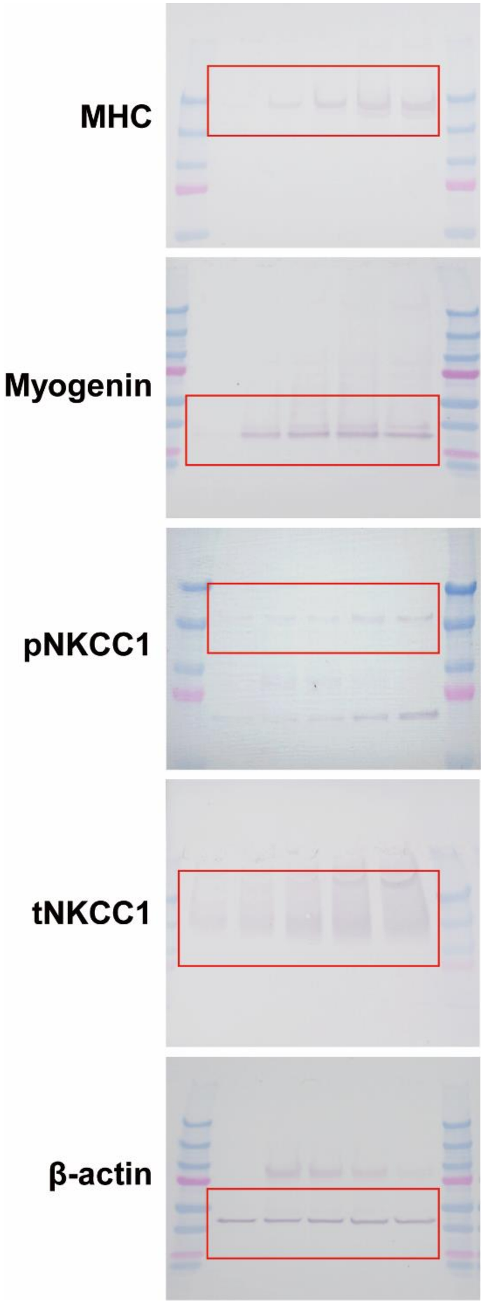

Original western blots for the images shown in Figure 1. The cropped images are highlighted in the red lines.

Supplementary Figure 8.

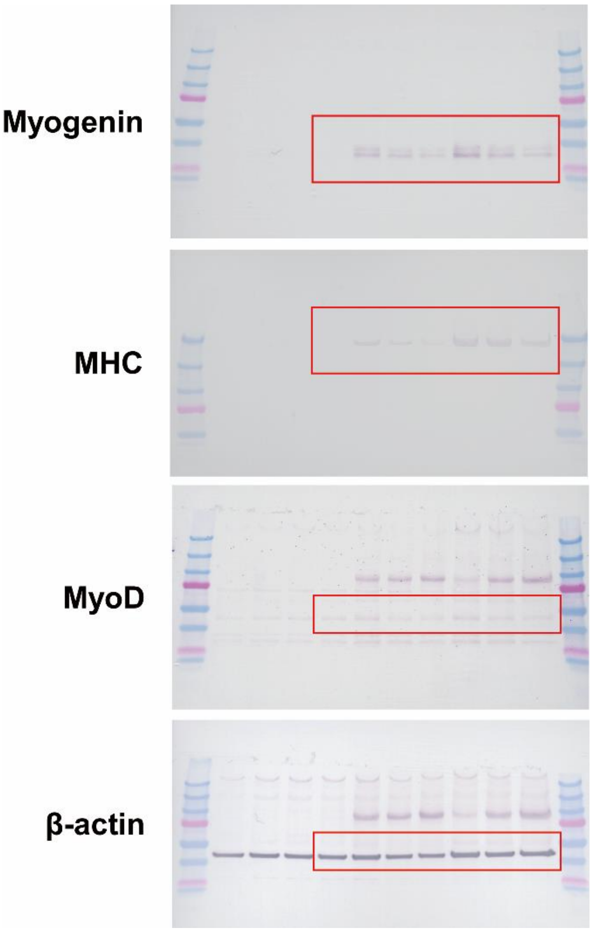

Original western blots for the images shown in Figure 2. The cropped images are highlighted in the red lines.

Supplementary Figure 9.

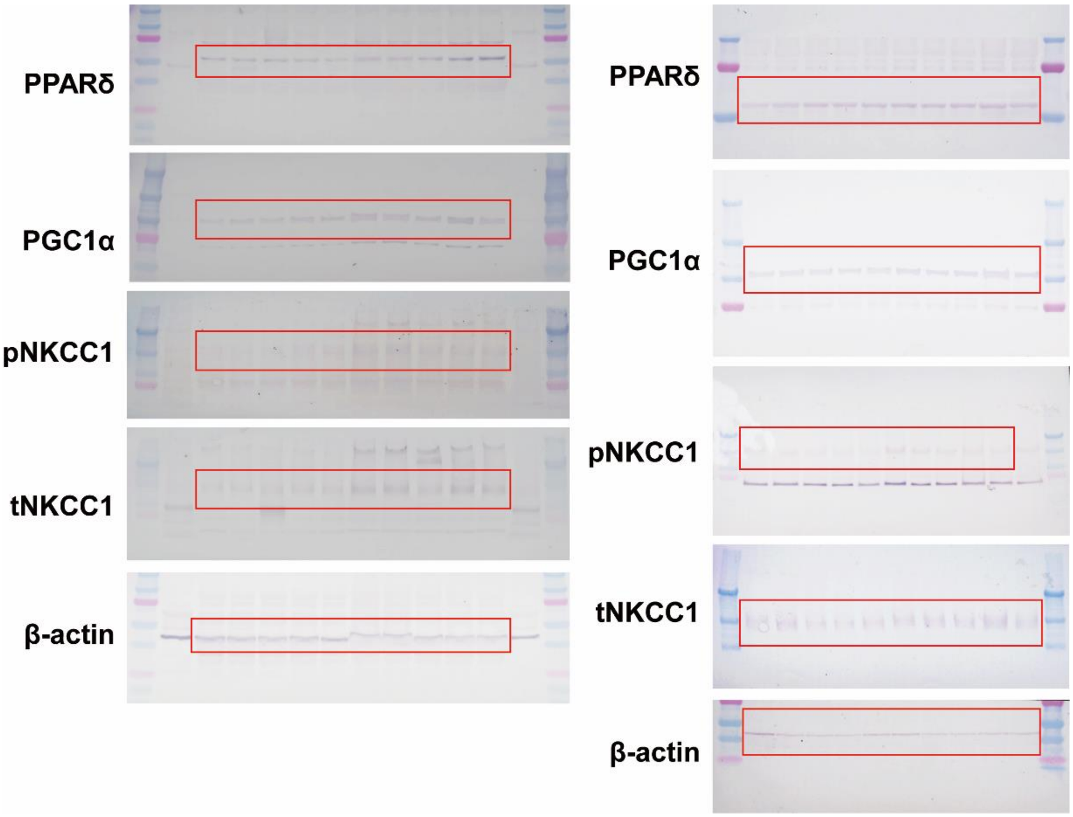

Original western blots for the images shown in Figure 3. The cropped images are highlighted in the red lines.

Supplementary Figure 10.

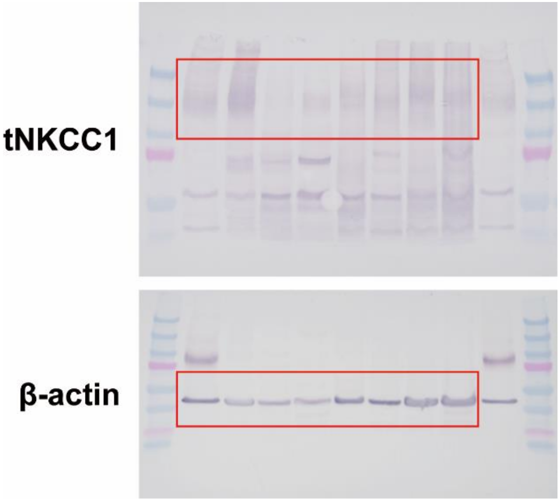

Original western blots for the images shown in Figure S6. The cropped images are highlighted in the red lines.

Supplementary Table 1. Nightly voluntary wheel running activity and differences in body weight between the Exercise and No Exercise groups

	No Exercise Group		Exercise Group	
Weeks	Body weight (g)	Distance (km)	Body weight (g)	Distance (km)
0	26.6 ± 0.7		26.2 ± 0.6	
3	28.0 ± 0.7	N/A	27.6 ± 0.6	3.0 ± 1.0
6	28.4 ± 0.7	N/A	28.8 ± 0.6	4.5 ± 1.4

Values are presented as the mean ± standard error of the mean (n = 5 per experimental group).

Supplementary Table 2. Primer sequences used for quantitative real-time polymerase chain reaction

Genes	Forward primer	Reverse primer
ACTB (β-actin)	CTCTGGCTCCTAGCACCATG	GTACTCCTGCTTGCTGATCC
Myh2 (MHC2A)	CGCAATGCAGAAGAGAAAGC	CCATGTTCTTCTTCATCCGC
MyoD1	GTGGCGACTCAGATGCATCC	TCACTGTAGTAGGCGGTGTC
Myog (myogenin)	GCCTCCTGCAGTCCGGAGTG	CGTGATGCTGTCCACGATGG

MHC2A, myosin heavy chain type IIa; Myh2, myosin heavy polypeptide 2; MyoD1, myogenic differentiation 1.
